# Exposure to environmental air pollutants as a risk factor for primary Sjögren’s syndrome

**DOI:** 10.3389/fimmu.2022.1044462

**Published:** 2023-02-14

**Authors:** Kevin Sheng-Kai Ma, Li-Tzu Wang, Weikun Chong, Cheng-Li Lin, Hailang Li, Aimin Chen, James Cheng-Chung Wei

**Affiliations:** ^1^ Department of Pediatrics, BenQ Medical Center, The Affiliated BenQ Hospital of Nanjing Medical University, Nanjing, Jiangsu, China; ^2^ Department of Epidemiology, Harvard T.H. Chan School of Public Health, Boston, MA, United States; ^3^ Center for Global Health, Perelman School of Medicine, University of Pennsylvania, Philadelphia, PA, United States; ^4^ Department of Orthodontics and Dentofacial Orthopedics, Henry M. Goldman School of Dental Medicine, Boston University, Boston, MA, United States; ^5^ Department of Obstetrics & Gynecology, National Taiwan University Hospital & College of Medicine, Taipei, Taiwan; ^6^ Clinical Trial Research Center, China Medical University Hospital, Taichung, Taiwan; ^7^ Department of Environmental Health, University of Cincinnati, Cincinnati, OH, United States; ^8^ Department of Biostatistics and Epidemiology, Perelman School of Medicine, University of Pennsylvania, Philadelphia, PA, United States; ^9^ Division of Allergy, Immunology and Rheumatology, Chung Shan Medical University Hospital, Taichung, Taiwan; ^10^ Institute of Medicine, Chung Shan Medical University, Taichung, Taiwan; ^11^ Graduate Institute of Integrated Medicine, China Medical University, Taichung, Taiwan

**Keywords:** air pollution, carbon monoxide, nitric oxide, methane, interleukin-6, Sjögren’s syndrome

## Abstract

**Background:**

Environmental etiology of primary Sjögren’s syndrome (pSS), an autoimmune disease, has been proposed. This study determined whether the exposure to air pollutants was an independent risk factor for pSS.

**Methods:**

Participants were enrolled from a population-based cohort registry. Daily average concentrations of air pollutants from 2000 to 2011 were divided into 4 quartiles. Adjusted hazard ratios (aHRs) of pSS for exposure to air pollutants were estimated in a Cox proportional regression model adjusting for age, sex, socioeconomic status, and residential areas. A subgroup analysis stratified by sex was conducted to validate the findings. Windows of susceptibility indicated years of exposure which contributed the most to the observed association. Ingenuity Pathway Analysis was used to identify underlying pathways of air pollutant-associated pSS pathogenesis, using Z-score visualization.

**Results:**

Two hundred patients among 177,307 participants developed pSS, with a mean age of 53.1 years at acumulative incidence of 0.11% from 2000 to 2011. Exposure to carbon monoxide (CO), nitric oxide (NO), and methane (CH4) was associated with a higher risk of pSS. Compared to those exposed to the lowest concentration level, the aHRs for pSS were 2.04 (95%CI=1.29-3.25), 1.86 (95%CI=1.22-2.85), and 2.21 (95%CI=1.47-3.31) for those exposed to high levels of CO, NO, and CH4, respectively. The findings persisted in the subgroup analysis, in which females exposed to high levels of CO, NO, and CH4 and males exposed to high levels of CO were associated with significantly great risk of pSS. The cumulative effect of air pollution on pSS was time-dependent. The underlying cellular mechanisms involved chronic inflammatory pathways including the interleukin-6 signaling pathway.

**Conclusion:**

Exposure to CO, NO, and CH4 was associated with a high risk of pSS, which was biologically plausible.

## 1 Introduction

Sjögren’s syndrome (SS) is an autoimmune disease in which the destruction of the epithelium of the exocrine glands, including the salivary glands and lacrimal glands, leads to the hallmark symptoms of sicca syndrome (1). Patients with SS may also present articular, cutaneous, pulmonary, and muscular complications, with symptoms that may plateau, worsen or go into remission (1). SS affects about 35 million people worldwide, with manifestations of significant fatigue, joint and muscle pain, impairing their quality of life, and the development of extra glandular manifestations, including non-Hodgkin’s lymphoma in about 30% to 50% of patients (2). The incidence of primary SS (pSS) is estimated to be 7 cases per 100,000 people per year in the general population with a female-to-male predominance of 9:1 (1). It is significantly higher in middle-aged women than in men as the prevalence rate is 60.82 cases per 100,000 residents with a female-male ratio of 10.72, and a mean onset age of 60.8 ± 15.2 years (3, 4). As with most autoimmune diseases, genetic, environmental, and hormonal factors are incorporated in SS pathogenesis (1). Although the pathogenic pathways underlying SS have yet been completely elucidated (5), studies have described that the etiology and pathogenesis of SS are related to genetic susceptibility to environmental factors, viral infection, vitamin D deficiency, smoking, endocrine alternations and the role of angiogenesis (6).

On the other hand, air pollution has been considered as one consequence of globalization involving industrial development, fossil fuel burning, and climate change. With the rising awareness of air pollution and environmentalism, how urban air pollution could negatively affect human health (7), as a constituent of the ecosystems in the ecotoxicological context, has been brought to the attention of healthcare professionals. Studies have provided evidence that air pollution is associated with enhanced incidence of cardiovascular and respiratory diseases (8). In terms of rheumatic inflammatory diseases, inhalation of air pollutants has been proposed to be an independent risk factor for systemic inflammatory responses, which is associated with the development and progression of autoimmune diseases, such as systemic autoimmune rheumatic diseases (SARD), juvenile idiopathic arthritis (JIA), and rheumatoid arthritis (RA) (9). However, it remains unclear whether the exposure to air pollutants is also a vital factor for the pathogenesis of SS. Therefore, the aim of the present population-based cohort study was to determine whether the exposure to air pollutants could be an environmental risk factor for pSS.

## 2 Materials and methods

### 2.1 Data source

With the National Health Insurance Research Database (NHIRD), covering 99% of the population of Taiwan, 25 million people, this population-based cohort study was conducted. The NHIRD consisted of data on reimbursement claims sourced from the National Health Insurance (NHI) program (10–12), a single-payer National Health Insurance (NHI) program which was instituted in 1995 and covered nearly 99% of Taiwan’s population by the end of 2008 (13). Outpatient visits, hospital admissions, prescriptions, procedures, and disease diagnostic records were retrieved from NHIRD (14–26).

Air pollution data including concentrations of carbon monoxide (CO), nitrogen monoxide (NO), and methane (CH_4_) were obtained from the Taiwan Air Quality-Monitoring Database (TAQMD). This database was released by a governmental agency, Taiwan Air Quality Monitoring Network, which collected daily ambient air pollution data from 78 community-based monitoring stations, available on a real-time basis since 1993. Pollutant data on particulate matter (PM)_10_ (μg/m^3^), PM_2.5_ (μg/m^3^), ozone (O_3_) (parts per million, ppm), CO (ppm), CH_4_ (ppm), sulfur dioxide (SO_2_, parts per billion, ppb), NO_2_ (ppb), and NO (ppb), were retrieved for their daily mean concentrations from January 1st, 2000 to December 31^th^, 2011. The endpoint of the follow-up period of this study was the diagnosis of pSS, or December 2011. The daily average exposed air pollutant concentrations for each participant were estimated using the Inverse Distance Weighting Method (IDW method). As one means of spatial interpolation, the IDW method was used to give the predicted values of unknown points by weighting the average of known points. In the present study, data on air pollution within two years prior to the diagnosis of pSS were accessed, with the IDW method used to estimate the concentrations of air pollutants based on values of the concentrations measured by monitoring stations surrounding the registered address of each participant, considering the distance between the monitoring station and the registered address of the participant.

### 2.2 Study population

Populations residing in areas with ambient air quality-monitoring stations were set as the study population. Among them, those with a history of SS before 2000 were excluded. All participants were traced from January 1^st^, 2000 until the endpoint of the study period. Confounding factors including age, gender, annual individual income, and urbanization levels where the participants lived, were adjusted. The urbanization level was defined following the National Health Research Institutes (NHRI) classification system, in which all residential areas were stratified into 7 urbanization levels considering their population density, resident education level, agricultural activity, and healthcare accessibility (physician number per 100,000 residents) (27). Due to the lack of participants from rural areas of levels 4-7, this study included areas of levels 1-4, with level 1 as the most urbanized and level 4 as the least urbanized. Annual incomes were classified into 4 groups: less than 5,760 USD, 5,760–7,320 USD, 7,320–8,400 USD, and over 8,400 USD.

### 2.3 Definition of exposure and outcome

The exposure to air pollution was defined using daily average concentrations of each air pollutant from 2000 to the endpoint. The daily average concentrations were classified into four groups using interquartile range (IQR), with three cut-off points (25th, 50th and 75th percentiles) ranging from the lowest concentration level, Quartile (Q) 1, to the highest concentration level, Q4. The American College of Rheumatology (ACR) and the European League Against Rheumatism (EULAR) Consensus Group criteria was followed to diagnose pSS as the outcome of this cohort study (28), for which the diagnosis of pSS was made based on anti-SSA (Ro) antibody positivity, focal lymphocytic sialadenitis, abnormal ocular staining, Schirmer’s tests, and unstimulated salivary flow rates (28). The diagnosis of pSS had to be confirmed in at least two outpatient visits or at least one hospitalization record within two years, all of which were peer-reviewed by a rheumatologist.

### 2.4 Statistical analyses

The Chi-squared test was used to determine the differences in age, sex, urbanization level, annual income, season, and the incidence of pSS. A multivariable Cox proportional hazard regression model adjusted for confounding factors was used to derive the association between the exposure to air pollutants and the risk of new-onset pSS. Incidence rates (IRs) and adjusted hazard ratios (aHRs) for new-onset pSS in participants exposed to each concentration level of air pollutants were derived. HRs for quartile levels Q2, Q3, and Q4, were compared to the reference level, Q1. All analyses were conducted with SAS version 9.4 (SAS Institute, Cary, NC, USA). A two-sided *P*-value less than 0.05 was considered statistically significant.

### 2.5 Bioinformatic analyses

Canonical Pathway Analysis (29–32) was conducted on Ingenuity Pathway Analysis (IPA) software (QIAGEN, Hilden, Germany) using differentially expressed (DE) RNA-seq data of airway epithelial cells exposed to coarse (n=3), fine (n=3), and ultrafine particulate matter (PM) (n=3) versus control group (n=3) obtained from the National Center for Biotechnology Information-Gene Expression Omnibus database (GEO; GSE7010), as well as parotid gland tissue from patients with pSS (n=3) versus healthy adults (n=3) obtained from GEO (GSE40611). Positive and negative z-scores indicated up-regulation and down-regulation of targeted pathways, respectively. Molecular Activation Prediction (MAP) was performed to model the mechanisms by which air pollutants may trigger or regulate the pathogenesis of pSS.

## 3 Results

177,307 residents with available data for both their exposure to air pollutants and electronic medical records were included in this study. The mean age of patients who developed pSS (N=200) was 53.1 years, with 60.5% over 50 years of age (N= 121) and 85% of patients were females (N=170). Among all included air pollutants, exposure to CO, NO, and CH_4,_ was significantly associated with higher risks of pSS.

### 3.1 Baseline characteristics and the incidence of pSS in individuals exposed to CO, NO, and CH_4_

Participants exposed to CO in Q1 level were the oldest, with a median age of 41.1 years, and exhibited the least incidence of pSS (0.07%). Participants exposed to Q3 level of CO had a younger median age of 38.7 years and exhibited the highest incidence of pSS (0.18%) (**Table 1**).

**Table 1:** Patient information.

Variables	N	%	N	%	N	%	N	%	p-value
CO	Q1 (<0.56 ppm)		Q2 (0.56-0.68 ppm)		Q3 (0.68-0.81 ppm)		Q4 (>0.81 ppm)		
N=177290	N=43942		N=45488		N=40036		N=47824		
Age (mean, SD*)	41.1±16		39.1±15.1		38.7±15.1		39.2±15.2		<0.001
Male sex	19791	45	20018	44	17848	44.6	20728	43.3	<0.001
Annual income (USD) ^†^									<0.001
< 5760	7071	16.1	7790	17.1	6978	17.4	8470	17.7	
5760−7320	12457	28.4	14969	32.9	14357	35.9	16058	33.6	
7320-8400	12914	29.4	10161	22.3	7144	17.8	9664	20.2	
≥ 8400	11500	26.2	12568	27.6	11557	28.9	13632	28.5	
Urbanization level^&^									<0.001
1 (highest)	9456	21.5	10748	23.6	15509	38.7	24923	52.1	
2	13690	31.2	20404	44.9	9811	24.5	13599	28.4	
3	7616	17.3	6848	15.1	9439	23.6	6374	13.3	
4 (lowest)	13180	30	7488	16.5	5277	13.2	2928	6.12	
New-onset Sjogren’s syndrome	29	0.07	42	0.09	71	0.18	58	0.12	<0.001
NO	Q1 (<5.16 ppb)		Q2 (5.16-8.58 ppb)		Q3 (8.58-11.5 ppb)		Q4 (>11.5 ppb)		
N=177307	N=43218		N=45009		N=39988		N=49092		
Age (mean, SD*)	41.0±	16	39.7±	15.5	37.7±	14.7	39.5±	15.2	<0.001
Male sex	19272	44.6	20280	45.1	17486	43.7	21358	43.5	<0.001
Annual income (USD) ^†^									<0.001
< 5760	7061	16.3	7924	17.6	6722	16.8	8606	17.5	
5760−7320	11945	27.6	15079	33.5	14806	37	16016	32.6	
7320-8400	13340	30.9	9550	21.2	7683	19.2	9314	19	
≥ 8400	10872	25.2	12456	27.7	10777	27	15156	30.9	
Urbanization level^&^									<0.001
1 (highest)	9025	20.9	7924	17.6	6722	16.8	8606	17.5	
2	15053	34.8	15079	33.5	14806	37	16016	32.6	
3	4684	10.8	9550	21.2	7683	19.2	9314	19	
4 (lowest)	14456	33.5	12456	27.7	10777	27	15156	30.9	
New-onset Sjogren's syndrome	35	0.08	45	0.1	53	0.13	67	0.14	0.04
CH_4_:	Q1 (<2.00 ppm)		Q2 (2.00-2.04 ppm)		Q3 (2.04-2.10 ppm)		Q4 (>2.10 ppm)		
N=133884	N=35349		N=30071		N=43474		N=24990		
Age (mean, SD*)	38.4±14.2		38.8±14.7		37.9±14.7		41.6±16.6		<0.001
Male sex	15724	44.5	12970	43.1	19132	44	11274	45.1	<0.001
Annual income (USD) ^†^									<0.001
< 5760	5831	16.5	5012	16.7	7436	17.1	4585	18.4	
5760−7320	12584	35.6	9808	32.6	15431	35.5	7495	30	
7320-8400	7544	21.3	5937	19.7	9013	20.7	6266	25.1	
≥ 8400	9390	26.6	9314	31	11594	26.7	6644	26.6	
Urbanization level^&^									<0.001
1 (highest)	10340	29.3	11091	36.9	16730	38.5	8745	35	
2	9293	26.3	9736	32.4	15284	35.2	7160	28.7	
3	9085	25.7	5472	18.2	6854	15.8	3881	15.5	
4 (lowest)	6631	18.8	3772	12.5	4606	10.6	5204	20.8	
New-onset Sjogren’s syndrome	39	0.11	14	0.05	38	0.09	59	0.24	<0.001

* Respiratory infection (n=6), Epstein-Barr virus infection (2), adenovirus infection (1), cervical lymphadenitis (1), Norovirus enteritis (1), Yersinia enteritis (1), urinary tract infection (1), urticaria (1).

^&^The urbanization level was categorized by the population density of the residential area into 4 levels, with level 1 being the most urbanized and level 4 being the least urbanized area.

^†^ The numbers were converted from New Taiwan Dollars to US Dollars. One New Taiwan Dollar equaled 0.03 US Dollar.

HV; Healthy Volunteer, NA; Not applecable, IVIG; Intravenous immunoglobulin, CAAs; Coronary artery abnormalities, ASA; acetylsalicylic acid, PSL; prednisolone, mPSL pulse; intravenous high-dose methylprednisolone pulse therapy, CsA; cyclosporin A, PE; plasma exchange, DEX; dexamethasone, Abs; antibiotics.

Participants exposed to NO in the Q1 level were the oldest, with the median age of 41.0 years; whereas those exposed to the Q3 level were the youngest, with a median age of 37.7 years. Compared with participants exposed to Q1, Q2, and Q3 levels of NO concentration, those exposed to Q4 level exhibited the highest incidence of pSS (0.14%), with the median age of 39.5 years (**Table 1**). Overall, the exposure to NO was significantly associated with the incidence of pSS (**Table 1**).

Participants exposed to CH_4_ in the Q4 level were the oldest, with a median age of 41.6 years. Participants exposed to Q4 level had the highest incidence of pSS (0.24%), compared with those exposed to lower concentration levels. More participants exposed to Q4 level of CH4 lived in level 1 urbanization areas (35.0%) (**Table 1**).

### 3.2 Incidence rates and risks of pSS in individuals exposed to CO, NO, and CH_4_

**Figure 1:**
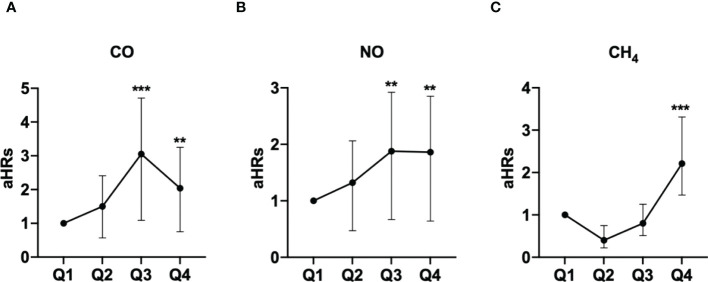
Illustrated hazard ratios (HRs) of pSS in individuals exposed to carbon monoxide (CO) **(A)**, nitric oxide (NO) **(B)**, and methane (CH_4_) **(C)**. **p<0.01; ***p<0.001.

The association between exposed levels of CO, NO, and CH_4_ and the incidence of pSS was dose-dependent. Subjects exposed to high concentrations (Q3 and Q4 levels) of CO, NO, and CH_4_, presented with significantly higher IRs of pSS, compared to those exposed to Q1 level of these air pollutants (**Table 2**). Likewise, after adjusting for demographics including age, sex, socioeconomic status, and urbanization levels of residing areas, the risk of pSS in those exposed to Q3 or Q4 levels were significantly higher than that in those exposed to to the Q1 level of each air pollutant (**Table 2** and **Figure 1**). In particular, the aHR was 3.05 (95% CI = 1.96–4.72) for individuals exposed to Q3 level of CO, 1.88 (95% CI = 1.21-2.92) for those exposed to Q3 level of NO, and 2.21 (95% CI = 1.47-3.31) for participants exposed to Q4 level of CH_4_ (**Table 2** and **Figure 1**).

**Table 2:** Incidence and risk of pSS in participants exposed to CO, NO, and CH_4_.

	Pollutant levels	Event	PY	IR	cHR	95%CI	aHR	95%CI
CO	N=177290							
Q1 (<0.56 ppm)	43942	29	508170	0.57	Ref.		Ref.	
Q2 (0.56-0.68 ppm)	45488	42	529881	0.79	1.39	(0.86, 2.22)	1.5	(0.93, 2.41)
Q3 (0.68-0.81 ppm)	40036	71	460849	1.54	2.7	(1.75, 4.16)***	3.05	(1.96, 4.71)***
Q4 (>0.81 ppb)	47824	58	549247	1.06	1.85	(1.19, 2.89)**	2.04	(1.29, 3.25)**
NO	N=177307							
Q1 (<5.16 ppb)	43218	35	500058	0.7	Ref.		Ref.	
Q2 (5.16-8.58 ppb)	45009	45	520789	0.86	1.24	(0.79, 1.92)	1.32	(0.85, 2.06)
Q3 (8.58-11.5 ppb)	39988	53	463131	1.14	1.63	(1.07, 2.50)*	1.88	(1.21, 2.92)**
Q4 (>11.5 ppb)	49092	67	564216	1.19	1.7	(1.13, 2.56)*	1.86	(1.22, 2.85)**
CH4	N=133884							
Q1 (<2.00 ppm)	35349	39	417257	0.93	Ref.		Ref.	
Q2 (2.00-2.04 ppm)	30071	14	354908	0.39	0.42	(0.23, 0.78)**	0.4	(0.22, 0.75)
Q3 (2.04-2.10 ppm)	43474	38	507527	0.75	0.8	(0.52, 1.26)	0.8	(0.51, 1.25)
Q4 (>2.10 ppm)	24990	59	275499	2.14	2.35	(1.57, 3.53)***	2.21	(1.47, 3.31)***

PY, person-years.

IR, incidence rate, (per 10,000 person-years).

cHR, crude hazard ratio.

aHR, adjusted hazard ratio obtained from a multivariate analysis, after adjusting for age, sex, annual income, and urbanization level.

CI, confidence interval.

Ref., reference group.

*p<0.05; **p<0.01; ***p<0.001.

**Figure 2:**
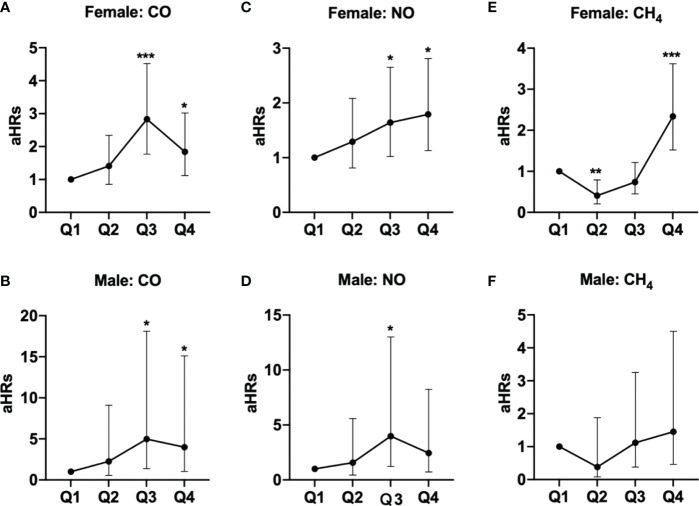
Illustrated hazard ratios (HRs) of pSS in females and males exposed to (**A**, female; **B**, male) CO, (**C**, female; **D**, male) NO, and (**E**, female; **F**, male) CH_4_. *p<0.05; ***p<0.001.

These findings persisted in a subgroup analysis stratified by sex, in which the risks of pSS among females exposed to Q3 (aHR =2.83, 95% CI = 1.77-4.52) and Q4 (aHR =1.84, 95% CI = 1.12-3.02) levels of CO, Q3 (aHR =1.64, 95% CI = 1.02-2.65) and Q4 (aHR =1.79, 95% CI = 1.13-2.81) levels of NO, and Q4 level of CH_4_ (aHR =2.34, 95% CI = 1.52-3.62), were significantly greater than females exposed to Q1 levels of respective air pollutants; parallelly, males exposed to Q3 (aHR =4.97, 95% CI = 1.37-18.1) and Q4 (aHR =3.99, 95% CI = 1.05-15.1) levels of CO also had significantly higher risks of pSS, compared to males exposed to Q1 level of CO (**Table 3** and **Figure 2**).

**Table 3:** Incidence and risk of pSS in participants exposed to CO, NO, and CH_4_, as stratified by sex.

	Pollutant levels	Event	PY	IR	cHR	95%CI	aHR	95%CI
Female								
CO	N=98905							
Q1 (<0.56 ppm)	24151	26	281533	0.92	Ref.		Ref.	
Q2 (0.56-0.68 ppm)	25470	36	298097	1.21	1.31	(0.79, 2.16)	1.41	(0.85, 2.34)
Q3 (0.68-0.81 ppm)	22188	60	257245	2.33	2.53	(1.60, 4.01)***	2.83	(1.77, 4.52)***
Q4 (>0.81 ppm)	27096	48	314121	1.53	1.66	(1.03, 2.67)*	1.84	(1.12, 3.02)*
NO	N=98911							
Q1 (<5.16 ppb)	23946	31	279250	1.11	Ref.		Ref.	
Q2 (5.16-8.58 ppb)	24729	39	288126	1.35	1.22	(0.76, 1.96)	1.29	(0.81, 2.08)
Q3 (8.58-11.5 ppb)	22502	42	262029	1.6	1.44	(0.91, 2.30)	1.64	(1.02, 2.65)*
Q4 (>11.5 ppb)	27734	58	321605	1.8	1.63	(1.63, 2.52)*	1.79	(1.13, 2.81)*
CH4	N=74784							
Q1 (<2.00 ppm)	19625	33	232163	1.42	Ref.		Ref.	
Q2 (2.00-2.04 ppm)	17101	12	202339	0.59	0.42	(0.22, 0.81)**	0.41	(0.21, 0.79)**
Q3 (2.04-2.10 ppm)	24342	30	285615	1.05	0.74	(0.45, 1.22)	0.74	(0.45, 1.22)
Q4 (>2.10 ppm)	13716	53	153862	3.44	2.47	(1.60, 3.82)***	2.34	(1.52, 3.62)***
Male								
CO	N=78385							
Q1 (<0.56 ppm)	19791	3	226637	0.13	Ref.		Ref.	
Q2 (0.56-0.68 ppm)	20018	6	231784	0.26	1.95	(0.49, 7.81)	2.26	(0.56, 9.09)
Q3 (0.68-0.81 ppm)	17848	11	203605	0.54	4.09	(1.14, 14.6)	4.97	(1.37, 18.1)*
Q4 (>0.81 ppm)	20728	10	235126	0.43	3.22	(0.89, 11.7)	3.99	(1.05, 15.1)*
NO	N=78396							
Q1 (<5.16 ppb)	19272	4	220809	0.18	Ref.		Ref.	
Q2 (5.16-8.58 ppb)	20280	6	232663	0.26	1.43	(0.40, 5.05)	1.57	(0.44, 5.58)
Q3 (8.58-11.5 ppb)	17486	11	201103	0.55	3.02	(0.96, 9.50)	3.98	(1.22, 13.0)*
Q4 (>11.5 ppb)	21358	9	242611	0.37	2.05	(0.63, 6.66)	2.44	(0.72, 8.24)
CH4	N=59100							
Q1 (<2.00 ppm)	15724	6	185094	0.32	Ref.		Ref.	
Q2 (2.00-2.04 ppm)	12970	2	152569	0.13	0.41	(0.08, 2.01)	0.38	(0.08, 1.88)
Q3 (2.04-2.10 ppm)	19132	8	221913	0.36	1.12	(0.39, 3.24)	1.12	(0.38, 3.25)
Q4 (>2.10 ppm)	11274	6	121637	0.49	1.58	(0.51, 4.91)	1.45	(0.46, 4.50)

PY, person-years.

IR, Incidence rate, (per 10,000 person-years).

cHR, crude hazard ratio.

aHR, adjusted hazard ratio of a multivariate analysis, after adjustment for age, sex, annual income, and urbanization level.

CI, confidence interval.

Ref., reference group.

*p<0.05; **p<0.01; ***p<0.001.

**Figure 3:**
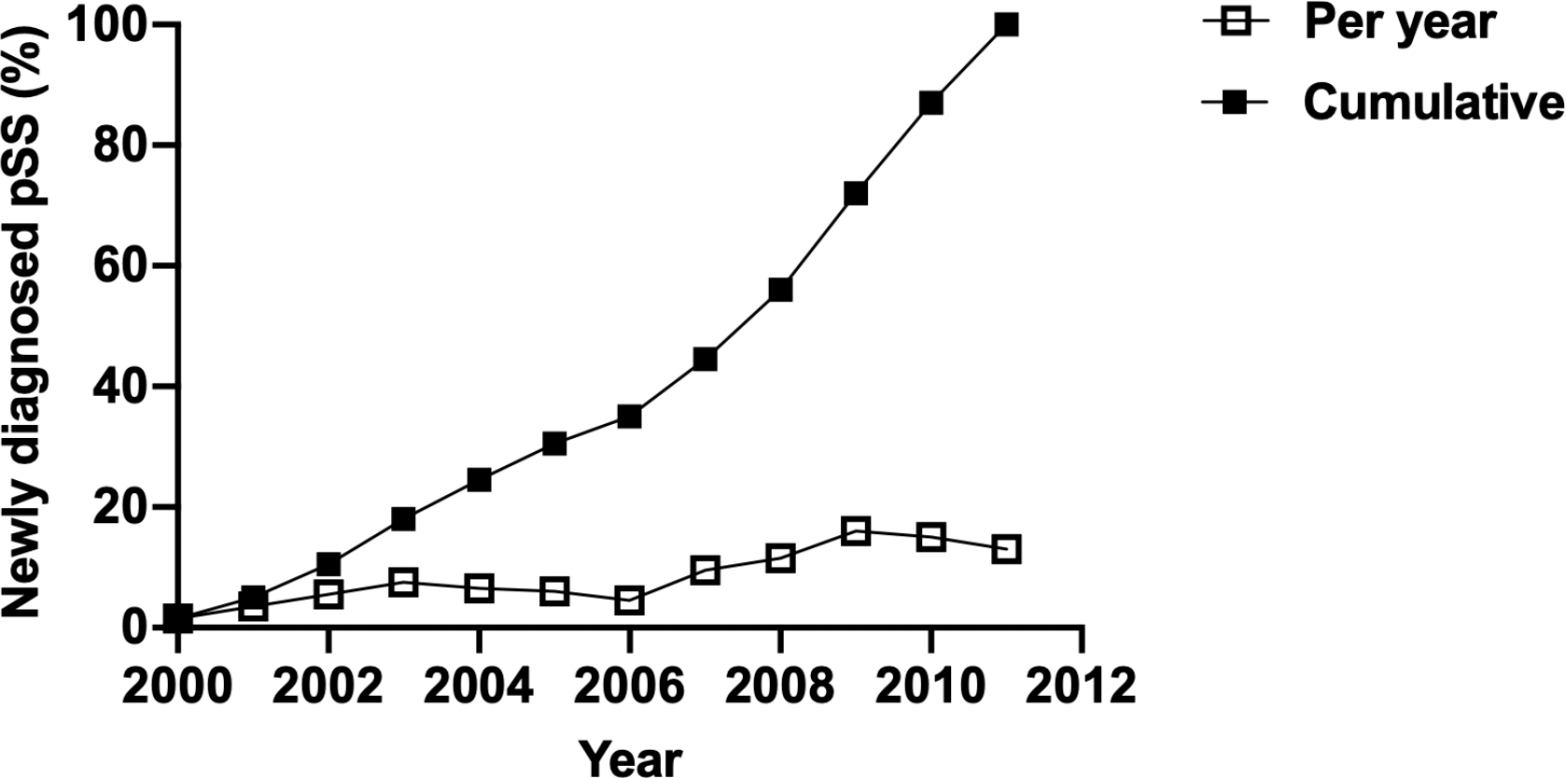
Incidence of newly diagnosed pSS per year and cumulative incidence of newly diagnosed pSS.

Overall, the dose-dependent effect of CO, NO, and CH_4_ on the risk of new-onset pSS was observed; on the other hand, the risks of pSS following the exposure to PM_10_, PM_2.5_, and NO_x_, were not consistently increased in individuals exposed to all high concentrations (**Supplementary Figure 1** and **Supplementary Table 1**). Collectively, these findings, suggested an elevated risk of pSS subsequent to the exposure to air pollutants that where small molecules, but not air pollutants of large sizes. Moreover, windows of susceptibility indicated years of exposure that contributed the most to the observed association, in which years between 2007 and 2011 were of high incidence of pSS (9.50%, 16.00%, and 13.00% of all incident cases of pSS happened in 2007, 2009, and 2011, respectively) (**Figure 3**). This suggested the time-dependent cumulative effect of the exposure to air pollutants on pSS.

### 3.3 Modeled mechanisms of air pollutant-triggered pSS

**Figure 4:**
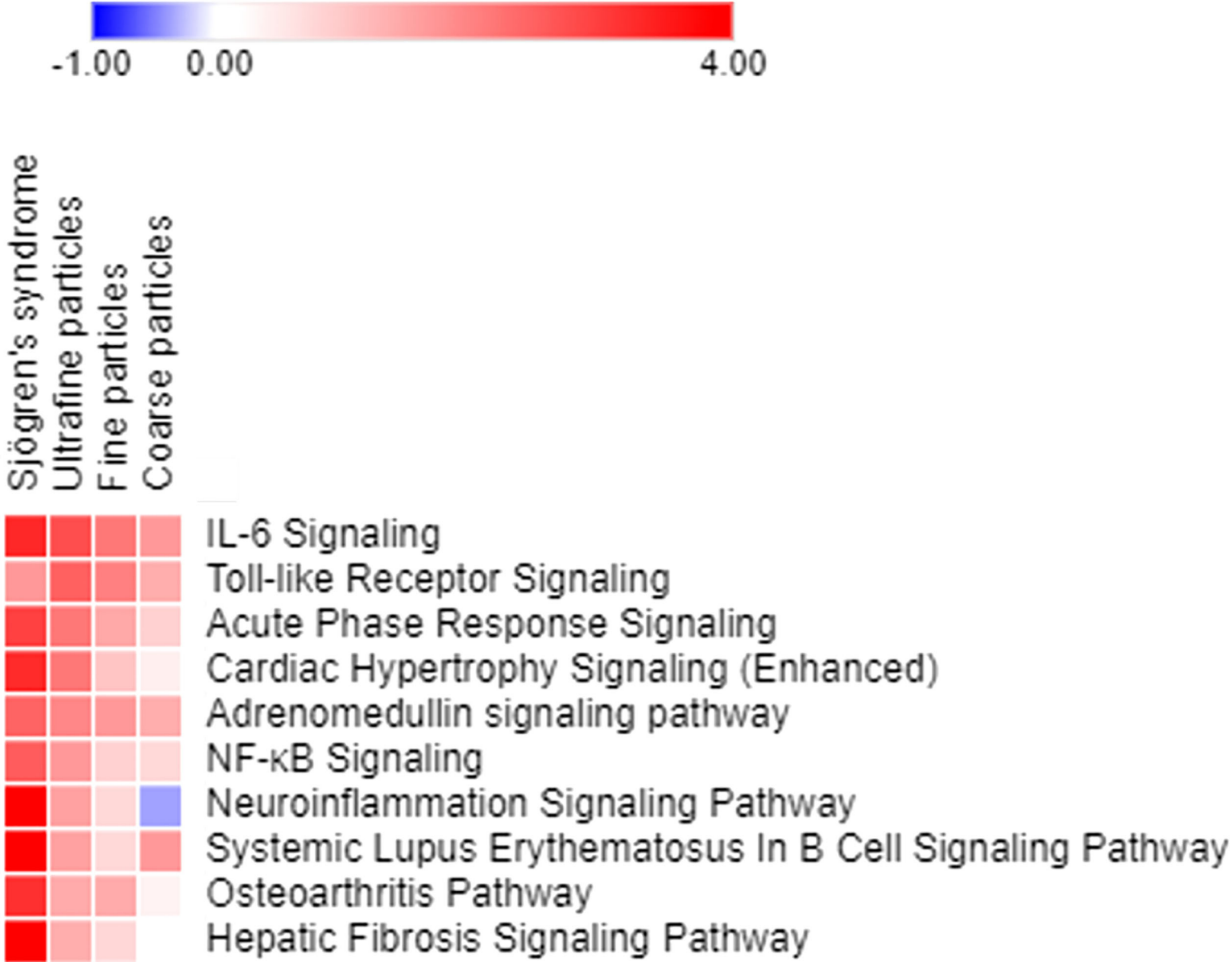
Pathways expressed in parotid glands of patients with pSS and primary airway epithelial cells exposed to ultrafine, fine, and coarse PM, ordered based on the sum of z scores.

Canonical pathway analyses indicated multiple air pollutant-associated inflammatory pathways that may trigger pSS, for which their degrees of upregulation were associated with particle sizes, with smaller sizes of air pollutants exacerbating larger extents of inflammation (**Figure 4**). Among pathways expressed in airway epithelial cells exposed to coarse, fine and ultrafine PM, and in parotid glands of patients with pSS, the highly expressed inflammatory pathways included (1): interleukin (IL)-6 signaling, with Z-scores being 3.272 for pSS, 2.714 for exposure to ultrafine PM, 2.121 for exposure to fine PM, and 1.663 for exposure to coarse PM, (2) Toll-like receptor signaling, with Z-scores being 1.633 for pSS, 2.449 for exposure to ultrafine PM, 2 for exposure to fine PM, and 1.342 for exposure to coarse PM, (3) acute phase response signaling, with Z-scores being 2.921 for pSS, 2.111 for exposure to ultrafine PM, 1.414 for exposure to fine PM, and 0.816 for exposure to coarse PM, (4) adrenomedullin signaling, with Z-scores being 2.401 for pSS, 1.89 for exposure to ultrafine PM, 1.633 for exposure to fine PM, and 1.342 for exposure to coarse PM, (5) NF-κB signaling, with Z-scores being 2.502 for pSS, 1.633 for exposure to ultrafine PM, 0.816 for exposure to fine PM, and 0.707 for exposure to coarse PM, (6) neuroinflammation signaling, with Z-scores being 4.899 for pSS, 1.508 for exposure to ultrafine PM, 0.707 for exposure to fine PM, and -0.378 for exposure to coarse PM, (7) B cell signaling pathway with Z-scores being 4.737 for pSS, 1.508 for exposure to ultrafine PM, 0.707 for exposure to fine PM, and 1.633 for exposure to coarse PM, and (8) osteoarthritis pathway with Z-scores being 3.157 for pSS, 1.387 for exposure to ultrafine PM, 1.387 for exposure to fine PM, and 0.333 for exposure to coarse PM.

**Figure 5:**
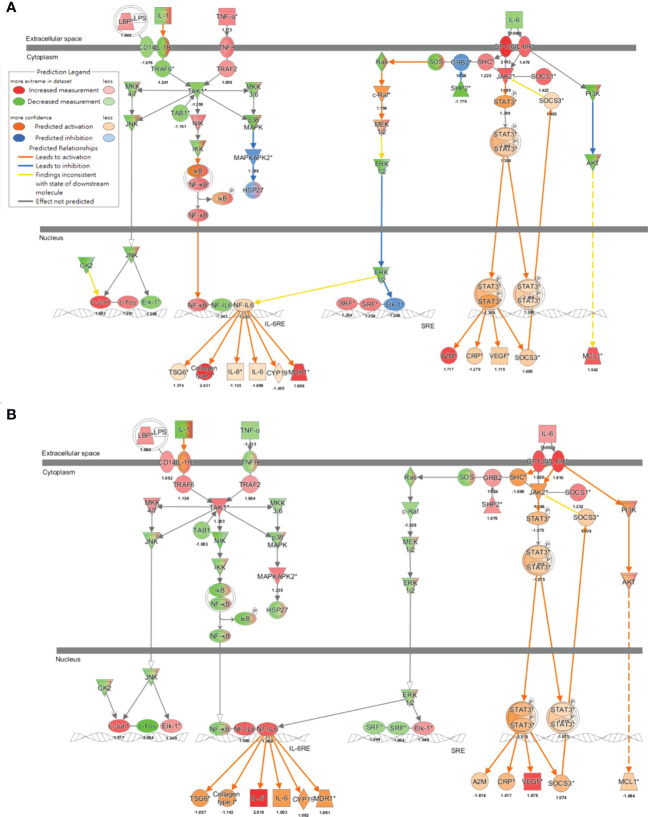
Air pollutant-associated IL-6 signaling pathway underlying the pathogenesis of pSS. Molecular Activation Prediction through transcriptomic data from parotid glands of patients with pSS **(A)** and airway epithelial cells exposed to ultrafine PM **(B)**, indicated mechanisms involved in IL-6 signaling.

To further decipher the network that addressed new-onset pSS following the exposure to air pollutants, findings of the MAP analysis (33, 34) supported that IL-6 signaling was activated in both the pathogenesis of pSS (**Figure 5A**) and the exposure to ultrafine (**Figure 5B**) or fine PM (**Supplementary Figure 1A**), in which transcription factor NF-IL6 was activated and subsequently induced the production of multiple inflammatory cytokines, including IL-6 and IL-8, while STAT3 was phosphorylated to transport downstream signaling and the production of acute-phase proteins, such as C-reactive protein (CRP). To the contrary, exposure to coarse PM only activated NF-IL6- but not STAT3-mediated signaling (**Supplementary Figure 1B**).

Overall, these inflammatory pathways, especially the IL-6 signaling, were upregulated more in response to the exposure to ultrafine particles than that to the exposure to coarse particles (**Figure 4**), which was in line with significant findings in the cohort study on small gaseous molecules.

## 4 Discussion

In this population-based cohort study, an independent correlation between exposure to small molecule air pollutants and new-onset pSS was observed. Individuals exposed to great concentrations of CH_4_, NO, CO, presented with significantly great risks of SS. After adjusting for age, sex, annual income, and urbanization levels of the areas where the participants lived, the association was still notable, for which our findings were not dominated by population density or socioeconomic status. The upregulated pathways in response to the exposure to air pollutants were proinflammatory, and their degrees of upregulation were related to PM sizes. Moreover, in accordance with the above-mentioned findings that small-molecule air pollutants, instead of PM_10_ or PM_2.5_, were associated with increased risks of pSS, the inflammatory pathways were most highly expressed in samples exposed to ultrafine particles, followed by those exposed to fine particles, then those exposed to coarse particles; these scale-dependent findings support the clinical relevance of findings in the present cohort study on significantly great risk of pSS following exposure to small molecule air pollutants (35).

Although previous studies suggested that socioeconomic status may affect the occurrence and development of autoimmune diseases (36), in the present study, the effect of air pollution on pSS was independent of socioeconomic status and where the participants lived. Moreover, results of canonical pathway analyses in the present study illustrated that such an association can be based on significantly triggered inflammatory pathways including the IL-6 signaling pathway that were highly expressed in airway epithelial cells and salivary glands. These findings were similar to previous studies suggesting that inhalation of air pollutants may directly affect the lungs, causing acute and chronic inflammation in the respiratory system (37), and may be associated with non-pulmonary diseases, such as type 2 diabetes, cancer, neurodegenerative diseases, and autoimmune rheumatoid diseases (38, 39). As such, together with previous studies (38, 39), findings of the present study allowed for the conjecture that air pollutants may not only directly induce local pulmonary inflammation but also trigger systemic chronic inflammatory responses, which was to the mechanisms by which air pollutants trigger cardiovascular diseases (8).

As to whether the exposure to air particulates could exacerbate or trigger SS, few studies have addressed the impact of airborne pollutants on SS. Most of these studies were either cross-sectional or animal studies. For instance, one animal study demonstrated that inhaled residual oil fly ash (ROFA) exacerbated lung response in SS mice models, mimicking the harmful effects of airborne pollution on the airway of patients with SS (40). Another cross-sectional study indicated that ocular abnormalities and eye irritation in patients with SS exposed to nitrogen dioxide were significantly more severe than control patients (41). These studies suggested a possible effect of air pollution on SS patients, yet did not provide evidence for whether the exposure to air pollutants was an independent risk factors for pSS, due to the lack of longitudinal and temporal data.

The respiratory immune system as the first-line defense against inhaled agents, when dysregulated by microorganisms, allergens or pollutants, has been known to trigger host immune responses (42) and autoantibody induction (43) that may initiate pSS (44). When these pollutants are transferred from lungs to blood, the onset of systemic inflammatory responses usually involves B cell stimulation, in which autoantibodies could be generated and lead to autoimmunity (38). In the present study, ambient exposure to severe air pollution involving CO, NO, and CH_4_ was associated with the risk of pSS, with previous studies suggesting that these three pollutants can trigger the production of excessive inflammatory mediators that may perturb immunity and cause pulmonary toxicity (45, 46). Juxtaposing findings in previous studies and the modeled air pollution-dependent pathogenesis of pSS as demonstrated in the present study, it is recognized that IL-6 plays a role in initiating Th17/Treg imbalance that may contribute to autoimmune responses and chronic inflammation, through driving Th17 immunity and inhibiting the peripheral generation of Foxp3^+^ regulatory T cells (47); furthermore, these IL-6-dependent autoimmune responses may be triggered by the exposure to air pollutants (34) and have been reported to be associated with the severity of pSS (48), providing the biological plausibility of findings in the present study. Specifically, epidemiological findings in the present study on significantly high risk of pSS following the exposure to air pollutants of smaller molecular sizes, such as CO, NO, and CH_4_, instead of PM_10_ and PM_2.5_, were in line with bioinformatic trends that as well indicated the biological effect of these pollutants on the triggered inflammatory pathways in airway epithelium, monocytes (49), and endothelium (50), which demonstrated a link between exposure to air pollutants and subsequent autoimmune responses.

Among all pSS-associated air pollutants that were identified in the present study, the findings on the correlation between pSS and the exposure to NO, echoed with studies suggesting that an increment of NO levels in the human body may disturb immune response, impair organelle function of alveolar macrophages, and elevate cellular levels of IFN-γ and MIP-1α, which can result in disrupted pulmonary immune homeostasis in the alveoli (51). As such, it is also worthwhile to uncover the mechanisms through which NO-mediated damage of alveolar macrophages may contribute to autoimmune pathogenesis underlying pSS, for which studies have suggested that epigenetic events in the immune cells can trigger pSS (6, 45), and that DNA methylation can be induced by exposure to particulate matter (52).

Furthermore, findings in the present study on the great risk of pSS in individuals exposed to high concentrations of air pollutants, were in line with studies implying the impact of environmental pollutants on dry eye disease in patients with SS and in SS models (53), for which increased levels of TNF-α and NF-κB in the cornea were observed (53, 54). Such an ocular involvement was followed by induced apoptosis in corneal superficial/basal epithelium that led to abnormal differentiation and proliferation of the ocular surface plus a reduced number of goblet cells in the conjunctival fornix (53), which was similar to our findings on air pollution-associated NF-κB upregulation. Additionally, since lung involvement as one of the major systemic complications of pSS (55) has been suggested to manifest as low transfer factors for carbon monoxide (55), our findings indicated that apart from dry eye (56–58) and dry mouth symptoms, the consecutive exposure to air pollutants may exacerbate pSS-related lung pathologies including chronic obstructive pulmonary disease and interstitial lung disease (55).

The strengths of the present cohort study included a large sample size with long-term follow-up, and the reliability of pSS as an outcome, as the diagnosis of pSS in this study was validated by rheumatologists, rather than questionnaires filled in by patients. Results of this study supported the hypothesis that individuals exposed to small molecule air pollutants, as opposed to PM_10_ or PM_2.5_, presented with an elevated risks of SS. The association between exposure to air pollutants and subsequent pSS may involve several chronic inflammatory pathways (59) with existing studies suggesting their relevance to autoimmune diseases such as the B cell signaling pathway (38). The findings on small molecule air pollutant-specific risk of pSS, were in accordance with scale-dependent expression levels of the above-mentioned chronic inflammatory pathways.

Although in the present study, people with previous history of pSS prior to the enrollment were excluded, there could be potential misclassification that resulted in cases who had undiagnosed pSS at baseline that were incorrectly found to develop pSS after the exposure to air pollutants. Moreover, as the exposure to air pollutants has been suggested to be associated with the risk of heart failure, stroke and myocardial infarction (60), it warrants further studies to investigate whether air pollution-associated pSS may be an early sign or concomitant complication of air pollution-associated heart diseases that could lead to hospitalization. Other limitations of this study included the lack of detailed information from electronic medical records involving lab data that may reflect the severity of SS, such as the raw data of SS disease activity index (ESSDAI) (1, 28). In addition, as the community-based monitoring stations of TAQMD may not detect indoor air quality, studies that collect data on indoor air pollutants are required to estimate the effect of indoor air quality; for instance, since NO can result from gas burning and barbecue or cooking (61), further studies that acquire data from NO sensors may validate findings in the present study. All in all, prospective cohort studies with more detailed information are needed to ascertain the observed dose-dependent effect of air pollutants on pSS and other autoimmune diseases, and to provoke patient education on environmental risk factors for pSS (62).

## 5 Conclusions

In conclusion, the exposure to small molecule air pollutants, especially CO, NO, and CH_4_, was an independent risk factor for pSS. Clinical and policy implications of this study include insights to environmental toxicology in a global health context, with more focus on ways to promote public awareness about the importance of environmental health to rheumatic and autoimmune diseases (13).

## Data availability statement

The datasets presented in this study can be found in online repositories. The names of the repository/repositories and accession number(s) can be found in the article/**Supplementary Material**.

## Author contributions

Conceptualization, KM, L-TW & WC; Methodology, KM, L-TW & C-LL; Writing – Original Draft, KM, HL & L-TW; Writing – Review & Editing, AC & JW; Funding Acquisition, KM, L-TW & WC. All authors contributed to the article and approved the submitted version.
