# Supplementary material for: Exposure to environmental air pollutants as a risk factor for primary Sjögren’s syndrome
Source: Front Immunol. 2023 Feb 14;13:1044462. doi: 10.3389/fimmu.2022.1044462 (PMC9972220; doi:10.3389/fimmu.2022.1044462)
Supplement: Supplementary file 1 [file DataSheet_1.docx]

Supplementary information

**Supplementary Table 1.** Incidence and risk of pSS in participants exposed to PM_10_, PM_2.5_, NO_x_, and NO_2_.

|  | Pollutant levels | Event | PY | IR | cHR | 95%CI | aHR | 95%CI |
| --- | --- | --- | --- | --- | --- | --- | --- | --- |
| PM_10_ |  |  |  |  |  |  |  |  |
| Q1(<51.6 μg/m^3^) | 51763 | 49 | 596573 | 0.82 | Ref. |  | Ref. |  |
| Q2(51.6-55.9 μg/m^3^) | 36222 | 50 | 420324 | 1.19 | 1.45 | (0.98, 2.15) | 1.53 | (1.03, 2.28)* |
| Q3(55.9-68.6 μg/m^3^) | 45108 | 66 | 523204 | 1.26 | 1.53 | (1.06, 2.22)* | 1.64 | (1.13, 2.39)** |
| Q4(≧68.6 μg/m^3^) | 44207 | 35 | 508095 | 0.69 | 0.84 | (0.54, 1.29) | 0.87 | (0.56, 1.35) |
| PM_2.5_ |  |  |  |  |  |  |  |  |
| Q1(<29.5 μg/m^3^) | 50056 | 31 | 586737 | 0.53 | Ref. |  | Ref. |  |
| Q2(29.5-33.3 μg/m^3^) | 38320 | 49 | 449580 | 1.09 | 2.06 | (1.32,.24)** | 2.12 | (1.35, 3.33)** |
| Q3(33.3-41.2 μg/m^3^) | 42115 | 46 | 493200 | 0.93 | 1.76 | (1.12, 2.78)* | 1.81 | (1.14, 2.87)* |
| Q4(≧41.2 μg/m^3^) | 43675 | 32 | 509569 | 0.63 | 1.19 | (0.73, 1.95) | 1.23 | (0.75, 2.03) |
| NO_x_ |  |  |  |  |  |  |  |  |
| Q1(<23.4 ppb) | 42060 | 38 | 486018 | 0.78 | Ref. |  | Ref. |  |
| Q2(23.4-32.0 ppb) | 47308 | 47 | 547209 | 0.86 | 1.1 | (0.72, 1.68) | 1.18 | (0.77, 1.81) |
| Q3(32.0-38.6 ppb) | 37218 | 57 | 430078 | 1.33 | 1.69 | (1.12, 2.55)* | 1.85 | (1.22, 2.81)** |
| Q4(≧38.6 ppb) | 50702 | 58 | 584821 | 0.99 | 1.27 | (0.84, 1.91) | 1.36 | (0.89, 2.07) |
| NO_2_ |  |  |  |  |  |  |  |  |
| Q1(<18.2 ppb) | 39202 | 35 | 453214 | 0.77 | Ref. |  | Ref. |  |
| Q2(18.2-23.7 ppb) | 51948 | 52 | 600458 | 0.87 | 1.12 | (0.73, 1.72) | 1.19 | (0.78 , 1.83) |
| Q3(23.7-27.5 ppb) | 52731 | 67 | 612227 | 1.09 | 1.41 | (0.94, 2.13) | 1.57 | (1.03, 2.40)* |
| Q4(≧27.5 ppb) | 33426 | 46 | 382296 | 1.2 | 1.56 | (1.01, 2.42)* | 1.71 | (1.08, 2.69)* |

PY= person-years.

IR= Incidence rate, (per 10,000 person-years).

cHR= crude hazard ratio.

aHR=adjusted hazard ratio of a multivariate analysis, after adjustment for age, sex, annual income, and urbanization level

CI= confidence interval.

Ref.= reference group

*p<0.05; **p<0.01; ***p<0.001

**Supplementary Figures**

**
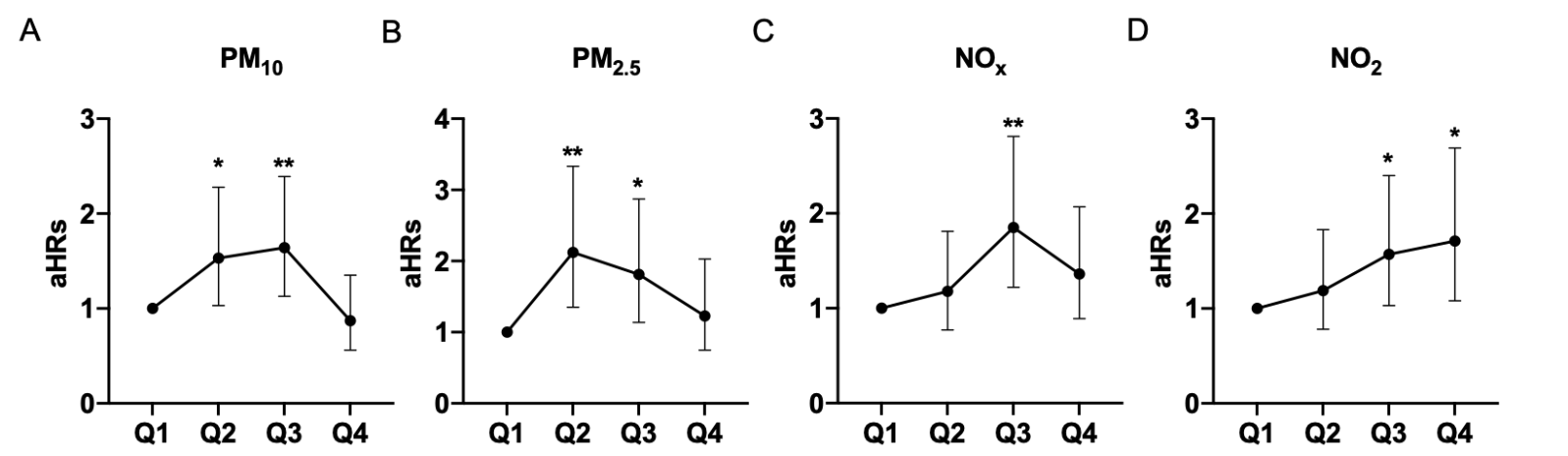
**

**Supplementary Figure 1.** Illustrated hazard ratios (HRs) of pSS in individuals exposed to particulate matter (PM)_10_ (A), PM_2.5_ (B), NOx (C), and NO_2_ (D).


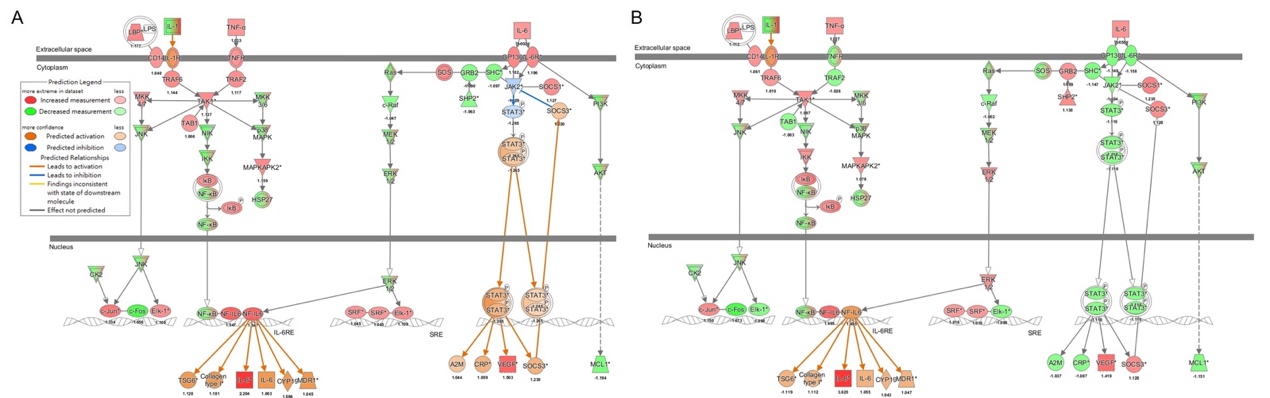


**Supplementary Figure 2.** Air pollutant-associated IL-6 signaling pathway underlying the pathogenesis of pSS. Molecular Activation Prediction through transcriptomic data from airway epithelial cells exposed to fine (A) and coarse PM (B), indicated mechanisms involved in IL-6 signaling.
